# Beneficial Effects of UV-Radiation: Vitamin D and beyond

**DOI:** 10.3390/ijerph13101028

**Published:** 2016-10-19

**Authors:** Christian Trummer, Marlene Pandis, Nicolas Verheyen, Martin R. Grübler, Martin Gaksch, Barbara Obermayer-Pietsch, Andreas Tomaschitz, Thomas R. Pieber, Stefan Pilz, Verena Schwetz

**Affiliations:** 1 Division of Endocrinology and Diabetology, Department of Internal Medicine, Medical University of Graz, Auenbruggerplatz 15, Graz 8036, Austria; christian.trummer@medunigraz.at (C.T.); marlene.pandis@medunigraz.at (M.P.); martin.gruebler@gmx.net (M.R.G.); martin.gaksch@gmail.com (M.G.); barbara.obermayer@medunigraz.at (B.O.-P.); thomas.pieber@medunigraz.at (T.R.P.); verena.schwetz@medunigraz.at (V.S.); 2 Division of Cardiology, Department of Internal Medicine, Medical University of Graz, Auenbruggerplatz 15, Graz 8036, Austria; nicolas.verheyen@medunigraz.at (N.V.); andreas.tomaschitz@medunigraz.at (A.T.); 3 Department of Cardiology, Swiss Cardiovascular Center Bern, Bern University Hospital, Freiburgstrasse 8, Bern 3010, Switzerland; 4 Bad Gleichenberg Clinic, Schweizereiweg 4, Bad Gleichenberg 8344, Austria

**Keywords:** vitamin D, 25(OH)D, UV-radiation, review

## Abstract

Aside from its well-known effects on bone and mineral metabolism, vitamin D may also play an important role in extra-skeletal processes like immunologic diseases, cancer, or cardiovascular diseases. Even though meta-analyses showed that vitamin D supplementation reduces fractures, falls, and overall mortality, its potential benefits did not find universal acclaim. Several health care authorities published Recommended Dietary Allowances (RDAs) for vitamin D, most of them ranging from 600 to 800 international units (IU) per day, corresponding to a serum level of 25-hydroxyvitamin D of at least 20 ng/mL (50 nmol/L). However, studies conducted in the general population revealed a much lower overall intake of vitamin D than the proposed RDAs. Thus, strategies to increase the vitamin D intake in the general population, e.g., food fortification or vitamin D supplementation, are needed to match the existing evidence and recommendations. Therefore, several currently ongoing projects aim to investigate the effect of vitamin D supplementation in the general population and try to establish food-based solutions to improve vitamin D status.

## 1. Introduction

Vitamin D ensures calcium supply and is thus essential for the maintenance of skeletal health. It increases calcium absorption in the gut and calcium reabsorption in the kidneys. Vitamin D deficiency due to malnutrition or low sunlight exposure causes impairment of bone mineralization resulting in skeletal deformities referred to as rickets [1,2,3]. Even before the discovery of vitamin D, it became evident that sunlight or ultraviolet B (UV-B) radiation, leading to vitamin D synthesis in the skin, was effective in treatment and prevention of rickets [1]. Nowadays, vitamin D supplementation in babies and infants is widely established as a measure to counteract the development of rickets [1,2,3], the prevalence of which has decreased since the mid 20th century in Western European countries [2]. In Africa, the Middle East and Asia, rickets still has a much higher prevalence and represents a major health burden [2]. In adults, vitamin D deficiency and potentially subsequent osteomalacia—a condition of muscle weakness and bone pain due to mineralization defects—is increasingly observed, especially with advancing age [4,5].

In recent years, along with the identification of the nearly ubiquitous expression of the vitamin D receptor and the discovery that almost all cells respond to 1,25-dihydroxyvitamin D (1,25(OH)_2_D) exposure, came the conviction that the effects of vitamin D could outreach those on skeletal health. The vitamin D receptor (VDR), a ligand-activated transcription factor, plays a role in the regulation of about 3% of the mouse or human genome [6], suggesting a more widespread function. Thus, vitamin D might be relevant for many extraskeletal diseases and for overall well-being [6,7,8]. In the liver, vitamin D is hydroxylated to its metabolite 25-hydroxyvitamin D (25(OH)D). In the kidneys, 25(OH)D is converted to 1,25(OH)_2_D, also referred to as the active vitamin D hormone calcitriol [6]. However, conversion of 25(OH)D to 1,25(OH)_2_D not only takes place in the kidneys, but also occurs in many extra-renal cells on a local level [6]. 25(OH)D can also be metabolized to 24,25(OH)_2_D by 25(OH)D-24-hydroxylase. 24,25(OH)D is considered a biologically inactive vitamin D metabolite and is thus destined for excretion [9]. Following a multitude of publications on vitamin D deficiency in the context of many chronic diseases, a discussion on the role of vitamin D supplementation in preventive medicine has emerged.

In this narrative review, we intended to outline existing evidence on the role of vitamin D deficiency in the classical context of osteoporosis, fractures and falls and on its role in extraskeletal health problems. Further, we give an overview of current data on the prevalence of vitamin D deficiency and the existing recommendations on supplementation. To conclude, we give an outlook on the ongoing trials regarding vitamin D deficiency and briefly highlight other UV-mediated effects aside from vitamin D.

## 2. Fractures, Falls and Vitamin D

Apart from its use in the prevention of rickets and osteomalacia, vitamin D is commonly accepted as a standard treatment in patients with osteoporosis [10]. In a pooled analysis of 11 randomized controlled trials (RCTs), patients with a mean age of 76 years receiving approximately 800 to 2000 International Units (IU) of vitamin D per day had a reduced risk of experiencing any non-vertebral or hip fracture [11]. Other meta-analyses have shown a reduction in hip or any type of fracture risk for vitamin D supplementation when administered with calcium [12] in institutionalized patients, but not in community-dwelling individuals [13].

Vitamin D has thus become a standard treatment for osteoporosis, albeit the mechanisms by which vitamin D reduces fracture risk have not been entirely elucidated, as vitamin D supplementation lacks a significant effect on bone mineral density (BMD) itself [14]. Widespread supplementation of vitamin D for primary osteoporosis prevention in community-dwelling adults without risk factors for vitamin D deficiency is thus under debate. The U.S. Preventive Services Task Force (USPSTF) recommends against daily supplementation with vitamin D for the primary prevention of fractures in noninstitutionalized postmenopausal women without a history of fractures [15]. However, individuals above the age of 65 are at increased risk of osteomalacia and fractures and are thus recommended to have a serum 25(OH)D level of at least 50 nmol/L (20 ng/mL), best achieved by supplementation of 800 IU per day, as suggested by a mainly European expert panel [16]. Bischoff-Ferrari et al. [11] have shown in an individual participant data meta-analysis that daily vitamin D doses of 800 to 2000 IU are required to achieve an anti-fracture effect, highlighting the still ongoing debate on dosage and frequency of vitamin D administration. 

Another possible mechanism underlying the antifracture effect of vitamin D could be the potential prevention of falls, as vitamin D deficiency has been associated with muscle weakness. Various meta-analyses on this topic, have, however, shown inconsistent results as to whether vitamin D supplementation actually reduces the risk of falls. The meta-analysis by Murad et al. has shown that vitamin D combined with calcium reduces the risk of falls [17], whereas pooled analyses by Bolland and colleagues show that supplementation with vitamin D, with or without calcium, does not reduce falls by 15% or more [18]. A very recent RCT shows that even though monthly vitamin D doses of 60,000 IU and 24,000 IU plus calcifediol were more likely than 24,000 IU to result in 25(OH)D levels of at least 30 ng/mL (75 nmol/L), no benefit on lower extremity function could be achieved by higher doses. Interestingly, higher doses were even associated with increased risk of falls as compared to 24,000 IU of vitamin D over a period of 12 months [19].

The divergent findings in the published meta-analyses on vitamin D and falls might be owed to methodological differences such as disparity in inclusion criteria and data extraction [20]. At present, the number of meta-analyses and systematic reviews outweighs the number of original RCTs. The disparity in conclusions drawn among overlapping meta-analyses gives rise to concern and constitutes a major task in the field of vitamin D requiring solution in the near future [20].

## 3. Cancer and Vitamin D

One of the first associations between sunlight exposure and cancer incidence dates back to 1941, when Apperly published his study showing a higher incidence of deaths from skin cancer in warmer U.S. states and Canadian provinces, but a lower incidence of other cancers, as well as an association between sun exposure (solar radiation index) and skin cancer mortality rates [21]. This study was an early basis for the discovery of the positive effect of sunlight—in part as a function of vitamin D—on cancer prevention [21], as later also postulated by Garland and Garland [22]. They proposed vitamin D as a protective factor against colon cancer development. As vitamin D production in the skin is dependent on UV-radiation, this hypothesis was derived from the geographic distribution of colon cancer in the U.S., linking the highest colon cancer mortality rates to regions with the least amounts of natural light [22]. Later epidemiological [23] as well as molecular studies support these early findings, the latter showing VDR activation to hamper initiation and progression of cancer [24,25]. Numerous epidemiological studies have shown inverse associations with levels of 25(OH)D and cancer risk at various sites [26,27,28]. Cancer survival in vitamin D deficient patients is lower than in patients without vitamin D deficiency [26,27,28,29]. Data from RCTs investigating the effect of vitamin D supplementation on cancer mortality have shown heterogeneous results, however. They largely failed to show a beneficial effect of vitamin D supplementation on cancer incidence and mortality [30], and, if so, only modest effects on overall cancer mortality but not on total cancer incidence could be observed [31,32]. However, the jury is still out on the subject matter due to limitations such as the high drop-out rate of studies analyzed in meta-analyses and due to restricted power of the existing vitamin D supplementation RCTs with primary end points that were mostly not cancer incidence or mortality as such [31,32]. Several ongoing studies aim to elucidate the effect of vitamin D supplementation on cancer, such as prostate cancer, colorectal cancer, thyroid cancer, breast cancer, etc.

However, it must be pointed out that UV-radiation is a proven carcinogen and is considered to be the main exogenous cause of cutaneous melanoma by the WHO [33]. General recommendations regarding a safe exposure of UV-radiation are nevertheless hard to give, since there exist significant individual differences concerning country or climate and skin type [34]. For white elderly people living in the Boston area, it has been estimated that the exposure of face, arms, and legs two to three times per week during the summer months for 5–10 min may be a sufficient strategy to optimize the cutaneous production of vitamin D [35,36]. Some authors recommended another approach taking the skin types described by Fitzpatrick [37] into account. According to these recommendations, adults living in a geographic latitude of 50–75 degrees N should expose a quarter of their body surface daily between 12:00 and 3:00 p.m. during the following times: June to August, skin type I 5–10 min, skin type II 10–15 min; March to May, skin type I 10–20 min, skin type II 15–25 min; September to October, skin type I 10–20 min, skin type II 15–25 min. These suggested exposure times can be doubled from 10:00 a.m. to 12:00 p.m. and 3:00 to 6:00 p.m. [38,39]. Diffey [40] invented a mathematical model to calculate changes in 25(OH)D serum levels from sun exposure. Estimations from this model came to the conclusion that, in the United Kingdom, a daily sun exposure of 10–20 min during the summer months leads to a maximum increase of 25(OH)D serum levels by 2–4 ng/mL (5–10 nmol/L). Of note, some groups like Vignali et al. [41] developed questionnaires to predict serum vitamin D levels based on sunlight exposure.

## 4. Extraskeletal Diseases and Vitamin D

Apart from its effects on skeletal health and is possible influence on cancer mortality, associations of vitamin D deficiency with many other chronic diseases have been reported, including autoimmune diseases, infections, cardiovascular and cerebrovascular diseases, and neurological diseases such as dementia [6,7,8,42,43,44,45,46,47,48]. According to an umbrella review on the vast number of existing systematic reviews and meta-analyses, strong evidence of a role of vitamin D is lacking for any outcome [45]. Only a few associations of vitamin D with a range of outcomes are probable according to the authors including birth weight, dental caries in children, maternal vitamin D concentrations at term, and parathyroid hormone (PTH) concentrations in patients with chronic kidney disease requiring dialysis [45]. Since the publication of the umbrella review, however, a meta-analysis on cardiovascular effects of vitamin D has shown that vitamin D supplementation might protect against cardiac failure in older people but not against myocardial infarction or stroke [49], implying that a clinically relevant protective cardiovascular effect of vitamin D cannot be excluded at present. As far as hypertension—considered one of the leading risk factors for global disease burden [50]—is concerned, studies have suggested that higher plasma concentrations of 25(OH)D may reduce risk of hypertension [51]. Some meta-analyses have suggested a blood pressure lowering effect of vitamin D supplementation [52,53], while other meta-analyses from 2015 could not confirm these findings and showed no effect of vitamin D supplementation on blood pressure [54,55]. Accordingly, in a recent RCT of 200 hypertensive patients no significant effect of vitamin D supplementation on 24 h-blood pressure could be observed [56]. Evidence also exists for vitamin D to play a role in fertility and steroidogenesis. However, randomized controlled trials are warranted to further investigate this possible effect [57,58,59]. In summary, again current evidence is scarce and definite conclusions on the role of vitamin D in non-skeletal diseases cannot be drawn at the moment; future studies might provide evidence on positive extraskeletal effects of vitamin D.

## 5. Mortality and Vitamin D

Many epidemiological studies have shown that vitamin D is independently associated with all-cause mortality not only in the general population [60,61,62], but also in patients with diabetes or the metabolic syndrome [63], in patients suffering from heart disease [64,65] or liver failure [66], in patients with chronic kidney disease [67] as well as in nursing home residents [68].

A meta-analysis of prospective cohort studies among general populations shows a nonlinear decrease in mortality risk with increasing levels of 25(OH)D [69]. According to a meta-analysis in patients with chronic kidney disease (CKD) [70], higher 25(OH)D levels are associated with significantly improved survival in these patients.

Optimal concentrations of 25(OH)D in terms of mortality risk range around 75–87.5 nmol/L (30–35 ng/mL) [69], as the association of vitamin D and mortality seems to follow a U- [71] or J-shaped curve [72].

Similar to the meta-analyses of observational studies, meta-analyses of RCTs have demonstrated an effect of vitamin D supplementation on mortality [13,32,73,74]. According to Bjelakovic and colleagues, for the prevention of one additional death, 150 individuals have to be treated for a period of five years [32]. As for the other described effects of vitamin D, limitations of the RCTs performed such as incomplete follow-up of data, warrant caution in the interpretation of results [13,32,73,74].

Despite these limitations, the available evidence on mortality and vitamin D deficiency to date has shown that vitamin D deficiency is not only a risk factor for increased mortality per se, but that vitamin D supplementation may reduce mortality. However, it is still unclear whether vitamin D itself should be considered as a cause for mortality or simply as an indicator of poor health [62].

Table 1 gives an overview over recent meta-analyses regarding vitamin D status or supplementation and health outcomes.

## 6. Reference Values for Dietary Intake of Vitamin D

Several countries and other health care authorities published recommendations regarding the dietary intake of vitamin D [75,76,77]. To a large extent, these recommendations match the report on dietary reference intakes for calcium and vitamin D updated by the Institute of Medicine (IOM) in 2010 [78]. Since the authors considered the evidence for extraskelatal effects of vitamin D to be inconsistent and insufficient, the intake recommendations were based on beneficial effects of vitamin D on skeletal health. Therefore, based on bone health, the following Recommended Dietary Allowances (RDAs) covering requirements of ≥97.5% of the population were proposed: 600 IU of vitamin D daily for ages 1–70 years and 800 IU daily for ages 71 years and older, corresponding to a 25(OH)D serum level of at least 20 ng/mL (50 nmol/L). Considering the wide variability in vitamin D synthesis from ultraviolet light and the associated risk of skin cancer, the RDAs apply to conditions of minimal or no sun exposure. It was further stressed that there is no consistent association of higher values with greater benefit. Additionally, U-shaped associations were observed for some clinical outcomes, indicating potential health risks at both low and high 25(OH)D levels [78].

It should be acknowledged that other organizations and health authorities recommended different RDAs: In their clinical practice guideline regarding evaluation, treatment and prevention of vitamin D deficiency, the Endocrine Society suggested that adults aged 19–50 years require at least 600 IU of vitamin D daily to maintain bone and muscular function. However, the task force further annotated that 1500–2000 IU per day are necessary to consistently raise the serum level of 25(OH)D above 30 ng/mL. Furthermore, different recommendations were given for certain populations including children (400 IU daily for children aged 0–1 years and 600 IU daily for older children), adults aged 50 years and older (600 IU daily for adults aged 50–70 years and 800 IU daily for adults aged 70 and older), and pregnant or lactating women (600 IU daily) [79]. For older adults aged 60 and above, the International Osteoporosis Foundation (IOF) recommended a RDA of 800–1000 IU in order to reach a serum 25(OH)D level of 30 ng/mL [80].

## 7. Definition and Prevalence of Vitamin D Deficiency

Since no universal cut-off or normal range for 25(OH)D serum levels exists, a general definition of vitamin D deficiency cannot be made. The proposed cut-offs for vitamin D status classification were subject to several scientific discussions on whether, e.g., 20 ng/mL (50 nmol/L) or 30 ng/mL (75 nmol/L) should be considered a sufficient level for 25(OH)D serum concentrations. However, there is broad agreement to prevent and treat 25(OH)D levels below 20 ng/mL (50 nmol/L). The IOM report on dietary reference intakes for calcium and vitamin D considers a 25(OH)D level of 20 ng/mL (50 nmol/L) to meet the requirement of 97.5% of the population and a level of 16 ng/mL (40 nmol/L) to meet the requirement of 50% of the population. Levels below 12 ng/mL (30 nmol/L) were found to indicate a risk for vitamin D deficiency [78].

A general consensus exists regarding the role of measurement of 25(OH)D to classify vitamin D status, since serum levels of 25(OH)D are considered to be the best parameter for vitamin D status reflecting vitamin D intake from all sources (i.e., dermal synthesis and dietary/supplemental intake). By contrast, measuring 1,25(OH)_2_D levels is not useful for monitoring vitamin D status, as it is tightly regulated by circulating levels of PTH, calcium, and phosphate and therefore does not adequately reflect vitamin D reserves [79].

Assessment of vitamin D status by measuring 25(OH)D can be achieved by a multitude of methodologies, including radioimmunoassay, high performance liquid chromotography, and liquid chromatography tandem mass spectroscopy. However, several significant differences between laboratories and assays exist [79,81,82,83,84]. Therefore, several attempts for standardization of 25(OH)D were made. This also led to the establishment of the Vitamin D Standardization Program (VDSP) by the National Institutes of Health (NIH) Office of Dietary Supplements in cooperation with the CDC National Center for Environmental Health (NCEH), the National Institute of Standards and Technology (NIST), and Ghent University in 2010 [85,86]. Nevertheless, several potential influences on 25(OH)D measurement, like the impact of certain vitamin D metabolites or the vitamin D binding protein (DBP), require further research [85,87].

Systematic reviews aiming to assess the prevalence of vitamin D deficiency on a global scale report a high prevalence of low 25(OH)D levels in the general population. In particular, a study by Wahl et al. [88] reported large regions around the globe with average 25(OH)D levels below 20 ng/mL (50 nmol/L). In another review by Hilger et al. [89], 37.5% of the included studies reported 25(OH)D levels below 20 ng/mL (50 nmol/L). Exploratory analyses revealed that newborns and institutionalized elderly individuals appear to be at a particular high risk of vitamin D deficiency. A summary of risk populations for vitamin D deficiency is given in Table 2.

Several studies also assessed vitamin D intake by nutrition and supplements. These studies reported a vitamin D intake from all sources in the general population below 200 IU per day, and therefore a much lower intake than the RDA [90,91].

## 8. Treatment of Vitamin D Deficiency

Vitamin D supply can be achieved from various sources, including cutaneous synthesis, food sources, and probably also by mobilization from vitamin D body stores. However, the impact of different sources on 25(OH)D levels is less clear and appears to be influenced by individual and environmental factors [76,77,92,93,94]. As reflected by the seasonal variation of 25(OH)D levels, cutaneous synthesis of vitamin D induced by UV-B appears to be the major source of 25(OH)D in most individuals. However, cutaneous vitamin D synthesis as well as vitamin D from other sources, like dietary intake, supplements, or mobilization from storages (e.g., adipose tissue) shows a high variation. Furthermore, levels of 25(OH)D and its metabolism are also regulated by certain hormones including PTH and fibroblast-growth-factor-23 (FGF-23) as well as inflammation [94,95]. Therefore, studies were unable to entirely explain variations in 25(OH)D levels, even if a comprehensive set of genetic, anthropometric, dietary, and lifestyle correlates was available [96].

Vitamin D intakes differ by age and gender as well as by country-specific vitamin D fortification practices. Supplements were identified as the most important determinant of variation in vitamin D intakes [92,97]. This is underlined by a study conducted by Black et al. [98], who reported a median daily intake of 348 IU of vitamin D in Irish adults using vitamin D supplements. In comparison, the median daily intake from usual diet was much lower (i.e., 84 IU of vitamin D). In North America, the median daily vitamin D intakes (from all sources) calculated from data of the National Health and Nutrition Examination Survey (NHANES) were 252 IU in children aged 2–18 years and was even lower for adults with 232 [99]. These circumstances highlight an obvious discrepancy between the actual intake of vitamin D and the proposed RDA. Therefore, strategies to increase vitamin D intake in the general population are needed to match the current evidence and recommendations.

Regarding the vitamin D status in the general population, the fortification of food with vitamin D has been considered to be the most promising strategy with the broadest reach and impact [77,92,98]. This has already been implemented in countries like the United States, Canada, or Finland and proved to have a significant influence on the daily vitamin D intake in the average adult [100]. In groups with high risk for vitamin D deficiency, vitamin D supplementation is arguably the most effective way to improve individual vitamin D status. Thus, expert panels considered a general vitamin D supplementation in populations at high risk for vitamin D deficiency [16].

Safety concerns regarding the dosage of vitamin D supplementation were raised by several observational studies. However, oral vitamin D intakes of up to 10,000 IU daily were not associated with any harmful effects. Characteristic symptoms of vitamin D intoxication, e.g., hypercalcemia, acute renal failure, and renal calcification, do not occur until serum levels of 25(OH)D are consistently above 150 to 200 ng/mL (375 to 500 nmol/L) [101]. Furthermore, an oral uptake of 10,000 IU vitamin D daily is comparable to the maximum cutaneous vitamin D production, and reports of vitamin D intoxication from cutaneous synthesis alone do not exist [101]. Since the dose response relationship between 25(OH)D and vitamin D intake shows a plateau at higher levels, very high intake doses of vitamin D are necessary to maintain toxic serum levels of 25(OH)D [102]. In this context, the IOM and the EFSA (European Food and Safety Authority) recommend a safe tolerable upper intake level of 4000 IU vitamin D per day for all adults including pregnant and lactating women [103]. However, very few data regarding the long-term effects of a vitamin D intake above the suggested threshold of 4000 IU per day are available [104,105].

Most experts recommend the use of vitamin D3 to treat and prevent vitamin D deficiency. This is partially based on some studies indicating an improved efficacy for vitamin D3 in raising serum 25(OH)D concentrations when compared to vitamin D2 [106]. A rule of thumb is that supplementation of 1000 IU of vitamin D3 daily leads to an approximate increase in 25(OH)D levels by 10–20 ng/mL (25–50 mmol/L), but there exists a significant inter-individual variation. This dose-response relationship is also highly dependent on other factors including baseline 25(OH)D level, body weight, and concomitant calcium intake [107,108,109]. After initiation of vitamin D supplementation, re-measurement of 25(OH)D serum levels should not be performed before at least two to three consecutive months of treatment, since some time is needed for 25(OH)D levels to reach a steady state [108].

Body weight possesses a significant impact on 25(OH)D serum levels. The most probable explanation derives from the lipophilic nature of vitamin D, leading to an increased deposit in adipose tissue in obesity and therefore lower circulating levels of 25(OH)D, but obese individuals also differ regarding sun exposure habits compared to lean persons. Several studies suggest that an increase in body mass index (BMI) leads to lower 25(OH)D concentrations, while effects of lower 25(OH)D do not provoke a rise in BMI in the same manner. Hence, population level interventions to support a reduction of body weight are expected to decrease the prevalence of vitamin D deficiency [110,111]. Overall, approximately one third of vitamin D deficiency may be attributable to obesity. Regarding vitamin D supplementation in obese subjects, some authors suggest that calcidiol might hypothetically be preferable to cholecalciferol due to its smaller volume of distribution. Furthermore, massive doses of cholecalciferol are oftentimes necessary to raise the serum level of 25(OH)D at least over 30 ng/mL [112,113].

## 9. Future Perspectives

It bears mentioning that in the existing literature almost all clinical studies defined vitamin D status by measuring serum levels of 25(OH)D. However, approximately 90% of circulating 25(OH)D is bound to DBP. Most studies investigating the effect of free vitamin D used mathematical models to estimate this component. These models rely on equations that utilize average binding coefficients for DBP and albumin and thus do not consider genotypic variations in binding affinity and/or serum concentration. Furthermore, studies reported inconsistent results regarding a possible correlation of free or bioavailable vitamin D with vitamin D-responsive response markers such as serum PTH levels. Therefore, further studies are required to assess the effect of free vitamin D [87,114,115,116].

Several vitamin D RCTs in the general population with large numbers of participants are currently ongoing and their results are expected to be published within the next few years. However, these trials are generally very costly and compromised by, e.g., compliance issues, as healthy subjects are more likely to ignore a regular intake of their study medication. Additionally, vitamin D trials are limited by the fact that participants in the control group—who usually should not receive any of the compound to be investigated—still produce vitamin D in their skin and consume it as part of their diet. Moreover, in several of the currently ongoing studies, subjects are allowed to keep taking low-dosed vitamin D supplements if they already did so before study entry [117,118].

Some projects, like the ODIN project (Food-based solutions for optimal vitamin D nutrition and health through the life cycle) funded by the European Union, aim to improve vitamin D status in the general population by development of food-based solutions [92,119]. This may be, apart from the skeletal health aspect, of special interest since some meta-analyses suggested a positive effect of vitamin D on survival by reducing all-cause and cancer mortality [32,69,72,74].

## 10. UV-Effects beyond Vitamin D

Several other effects aside from vitamin D synthesis are induced by exposition to UV radiation. The human skin is the largest storage of nitric oxide (NO) and its derivatives. Irradiation with relevant doses of UV-A induces a translocation of NO into the circulation, leading to vasodilatation, reduced vascular resistance, and a reduction in blood pressure [34,120,121,122]. UV radiation may lead to a degradation of folic acid, possibly leading to folate deficiency. As folic acid is involved in DNA synthesis and repair as well as amino acid metabolism, its presence is especially important in pregnant women and it has been proposed to play a significant role in carcinogenesis. Nevertheless, the consequences of this photodegradation on individual health remain unclear and require further research [34,123,124].

Serotonin levels are influenced by sunlight exposure of the skin and the eyes. Serotonin is a neurotransmitter that modulates mood, anxiety, aggression, pain, sexual behavior, and sleep [125]. Additionally, studies reported a risk-lowering effect of serotonin on diabetes mellitus and a risk-increasing effect on arterial hypertension [34,126,127]. Exposure to light also affects melatonin secretion. It is usually secreted at a daily rhythm, with low concentrations during daytime and a peak around midnight. The phase and the amplitude of the peak can be influenced by the amount of exposure to light during daytime. Studies reported anti-oxidant effects of melatonin as well as possible anti-metastatic and anti-angiogenic effects [34,125,128].

However, intermittent sun exposure, especially at a young age, is the main risk factor for development of melanoma [129]. Intermittent sun exposure is also an important risk factor for basal cell carcinoma and squamous cell carcinoma [130,131].

## 11. Conclusions

Vitamin D is mostly recognized for its effects on bone health and metabolism and is associated with several chronic diseases, including immunologic and cardiovascular diseases and cancer. However, it is still unclear whether vitamin D may positively influence the outcome of these extraskeletal processes. Humans may gain vitamin D from various sources, although UV-mediated cutaneous synthesis appears to have the biggest impact in most individuals. Recommendations for dietary intake of vitamin D are based on its effect on skeletal health. Nevertheless, a discrepancy between recommended RDAs and actual daily vitamin D intake exists. Therefore, solutions like food fortification or vitamin D supplementation in risk populations need to be developed. Apart from vitamin D, UV-radiation induces several other effects, including increased NO circulation and folic acid degradation.

## Figures and Tables

**Table 1:** Overview of selected recent meta-analyses of vitamin D status or supplementation and health outcomes (i.e., fractures and falls, cancer incidence and mortality, cardiovascular mortality).

Meta-Analyses	Biomarker	Meta-Analysis Metric	No. of Patients/Studies	No. of Events	Relative Risk	95% CI	*p*-Value
**Fractures and falls**							
Bischoff-Ferrari H. 2012 [11]	25(OH)D	HR	31,022 patients	1111 incident hip fractures	hip fracture: 0.90	hip fracture: 0.80, 1.01;	NR
				3770 nonvertebral fractures	nonvertebral fracture: 0.93	nonvertebral fracture: 0.87, 0.99	
Murad M.H. 2011 [17]	25(OH)D	OR	45,782 patients		OR for suffering at least one fall 0.86	0.77, 0.96	NR
**Cancer incidence and cancer mortality**							
Yin L. 2013 [28]	25(OH)D	RR	5 studies		total cancer incidence 0.89	0.81, 0.97	0.01
			13 studies		total cancer mortality 0.83	0.71, 0.96	<0.01
Keum N. 2014 [31]	25(OH)D	RR	4 RCTs, 45,151 participants	4333 cases	total cancer incidence 1.00	0.94, 1.06	0.998
			3 RCTs, 44,260 participants	1190 deaths	total cancer mortality 0.88	0.78, 0.98	0.02
**Cardiovascular mortality**							
Chowdhury R. 2014 [73]	25(OH)D	RR	29 studies, 101,649 participants	10,203 events	Cardiovascular mortality 1.43	1.25, 1.64	0.96

25(OH)D = 25-hydroxyvitamin D; CI = confidence interval; HR = hazard ratio; NR = not reported; OR = odds ratio; RCTs = randomized controlled trials; RR = relative risk.

**Table 2:** Vulnerable populations for vitamin D deficiency **(**adapted from the Endocrine Society clinical practice guidelines [79]).

Vulnerable Populations for Vitamin D Deficiency—Whom to Screen?
Patients with rickets and osteomalacia
Patients with osteoporosis
Patients with chronic kidney disease
Patients with hepatic failure
Patients with malabsorption syndromes
Patients with cystic fibrosis
Patients with inflammatory bowel disease
Patients with bariatric surgery
Patients with radiation enteritis
Patients with hyperparathyroidism
Patients with lymphomas
Patients with medications including antiseizure medications, glucocorticoids, anti-retroviral medications, antifungals, cholestyramine
African-American and Hispanic individuals
Pregnant and lactating women
Older adults with history of falls
Older adults with history of nontraumatic fractures
Obese individuals
Patients with granuloma-forming disorders, including sarcoidosis, tuberculosis, etc.

## References

[1] Holick M.F. (1994). McCollum Award Lecture, 1994: Vitamin D—New horizons for the 21st century. Am. J. Clin. Nutr..

[2] Prentice A. (2013). Nutritional rickets around the world. J. Steroid Biochem. Mol. Biol..

[3] Winzenberg T., Jones G. (2013). Vitamin D and bone health in childhood and adolescence. Calcif. Tissue Int..

[4] Lips P. (2001). Vitamin D deficiency and secondary hyperparathyroidism in the elderly: Consequences for bone loss and fractures and therapeutic implications. Endocr. Rev..

[5] Bhan A., Rao A.D., Rao D.S. (2012). Osteomalacia as a result of vitamin D deficiency. Rheum. Dis. Clin. N. Am..

[6] Bouillon R., Carmeliet G., Verlinden L., van Etten E., Verstuyf A., Luderer H.F., Lieben L., Mathieu C., Demay M. (2008). Vitamin D and human health: Lessons from vitamin D receptor null mice. Endocr. Rev..

[7] Pludowski P., Holick M.F., Pilz S., Wagner C.L., Hollis B.W., Grant W.B., Shoenfeld Y., Lerchbaum E., Llewellyn D.J., Kienreich K. (2013). Vitamin D effects on musculoskeletal health, immunity, autoimmunity, cardiovascular disease, cancer, fertility, pregnancy, dementia and mortality—A review of recent evidence. Autoimmun. Rev..

[8] Rosen C.J., Adams J.S., Bikle D.D., Black D.M., Demay M.B., Manson J.E., Murad M.H., Kovacs C.S. (2012). The nonskeletal effects of vitamin D: An endocrine society scientific statement. Endocr. Rev..

[9] Holick M.F., Schnoes H.K., DeLuca H.F., Gray R.W., Boyle I.T., Suda T. (1972). Isolation and identification of 24, 25-dihydroxycholecalciferol, a metabolite of vitamin D made in the kidney. Biochemistry.

[10] Kanis J.A., McCloskey E.V., Johansson H., Cooper C., Rizzoli R., Reginster J.Y. (2013). European guidance for the diagnosis and management of osteoporosis in postmenopausal women. Osteoporos. Int..

[11] Bischoff-Ferrari H.A., Willett W.C., Orav E.J., Lips P., Meunier P.J., Lyons R.A., Flicker L., Wark J., Jackson R.D., Cauley J.A. (2012). A pooled analysis of vitamin D dose requirements for fracture prevention. N. Engl. J. Med..

[12] Avenell A., Mak J.C., O’Connell D. (2014). Vitamin D and vitamin D analogues for preventing fractures in post-menopausal women and older men. Cochrane Database Syst. Rev..

[13] Bolland M.J., Grey A., Gamble G.D., Reid I.R. (2014). The effect of vitamin D supplementation on skeletal, vascular, or cancer outcomes: A trial sequential meta-analysis. Lancet Diabetes Endocrinol..

[14] Reid I.R. (2015). Effects of vitamin D supplements on bone density. J. Endocrinol. Investig..

[15] Moyer V.A. (2013). Vitamin D and calcium supplementation to prevent fractures in adults: U.S. preventive services task force recommendation statement. Ann. Intern. Med..

[16] Brouwer-Brolsma E.M., Bischoff-Ferrari H.A., Bouillon R., Feskens E.J., Gallagher C.J., Hypponen E., Llewellyn D.J., Stoecklin E., Dierkes J., Kies A.K. (2013). Vitamin D: Do we get enough? A discussion between vitamin D experts in order to make a step towards the harmonisation of dietary reference intakes for vitamin D across Europe. Osteoporos. Int..

[17] Murad M.H., Elamin K.B., Elnour N.O.A., Elamin M.B., Alkatib A.A., Fatourechi M.M., Almandoz J.P., Mullan R.J., Lane M.A., Liu H. (2011). Clinical review: The effect of vitamin D on falls: A systematic review and meta-analysis. J. Clin. Endocrinol. Metab..

[18] Bolland M.J., Grey A., Gamble G.D., Reid I.R. (2014). Vitamin D supplementation and falls: A trial sequential meta-analysis. Lancet Diabetes Endocrinol..

[19] Bischoff-Ferrari H.A., Dawson-Hughes B., Orav E.J., Staehelin H.B., Meyer O.W., Theiler R., Dick W., Willett W.C., Egli A. (2016). Monthly high-dose vitamin D treatment for the prevention of functional decline: A randomized clinical trial. JAMA Intern. Med..

[20] Bolland M.J., Grey A., Reid I.R. (2014). Differences in overlapping meta-analyses of vitamin D supplements and falls. J. Clin. Endocrinol. Metab..

[21] Apperly F.L. (1941). The relation of solar radiation to cancer mortality in North America. Cancer Res..

[22] Garland C.F., Garland F.C. (1980). Do sunlight and vitamin D reduce the likelihood of colon cancer?. Int. J. Epidemiol..

[23] Grant W.B. (2013). Update on evidence that support a role of solar ultraviolet-B irradiance in reducing cancer risk. Anticancer Agents Med. Chem..

[24] Chiang K.C., Chen T.C. (2013). The anti-cancer actions of vitamin D. Anticancer Agents Med. Chem..

[25] Höbaus J., Thiem U., Hummel D.M., Kallay E. (2013). Role of calcium, vitamin D, and the extrarenal vitamin D hydroxylases in carcinogenesis. Anticancer Agents Med. Chem..

[26] Pilz S., Tomaschitz A., Obermayer-Pietsch B., Dobnig H., Pieber T.R. (2009). Epidemiology of vitamin D insufficiency and cancer mortality. Anticancer Res..

[27] Pilz S., Kienreich K., Tomaschitz A., Ritz E., Lerchbaum E., Obermayer-Pietsch B., Matzi V., Lindenmann J., Marz W., Gandini S. (2013). Vitamin D and cancer mortality: Systematic review of prospective epidemiological studies. Anticancer Agents Med. Chem..

[28] Yin L., Ordonez-Mena J.M., Chen T., Schottker B., Arndt V., Brenner H. (2013). Circulating 25-hydroxyvitamin D serum concentration and total cancer incidence and mortality: A systematic review and meta-analysis. Prev. Med..

[29] Mohr S.B., Gorham E.D., Kim J., Hofflich H., Garland C.F. (2014). Meta-analysis of vitamin D sufficiency for improving survival of patients with breast cancer. Anticancer Res..

[30] Lazzeroni M., Serrano D., Pilz S., Gandini S. (2013). Vitamin D supplementation and cancer: Review of randomized controlled trials. Anticancer Agents Med. Chem..

[31] Keum N., Giovannucci E. (2014). Vitamin D supplements and cancer incidence and mortality: A meta-analysis. Br. J. Cancer.

[32] Bjelakovic G., Gluud L.L., Nikolova D., Whitfield K., Krstic G., Wetterslev J., Gluud C. (2014). Vitamin D supplementation for prevention of cancer in adults. Cochrane Database Syst. Rev..

[33] Solar and ultraviolet radiation (1992). IARC monographs on the evaluation of carcinogenic risks to humans. IARC.

[34] Van der Rhee H., de Vries E., Coomans C., van de Velde P., Coebergh J.W. (2016). Sunlight: For better or for worse? A review of positive and negative effects of sun exposure. Cancer Res. Front..

[35] Holick M.F. (1995). Environmental factors that influence the cutaneous production of vitamin D. Am. J. Clin. Nutr..

[36] Barger-Lux M.J., Heaney R.P. (2002). Effects of above average summer sun exposure on serum 25-hydroxyvitamin D and calcium absorption. J. Clin. Endocrinol. Metab..

[37] Fitzpatrick T.B. (1988). The validity and practicality of sun-reactive skin types I through VI. Arch. Dermatol..

[38] Holick M.F., Jenkins M. (2009). The UV Advantage.

[39] German Nutrition Society (2012). New reference values for vitamin D. Ann. Nutr. Metab..

[40] Diffey B.L. (2010). Modelling the seasonal variation of vitamin D due to sun exposure. Br. J. Dermatol..

[41] Vignali E., Macchia E., Cetani F., Reggiardo G., Cianferotti L., Saponaro F., Marcocci C. (2016). Development of an algorithm to predict serum vitamin D levels using a simple questionnaire based on sunlight exposure. Endocrine.

[42] Holick M.F., Binkley N.C., Bischoff-Ferrari H.A., Gordon C.M., Hanley D.A., Heaney R.P., Murad M.H., Weaver C.M. (2012). Guidelines for preventing and treating vitamin D deficiency and insufficiency revisited. J. Clin. Endocrinol. Metab..

[43] Rosen C.J., Abrams S.A., Aloia J.F., Brannon P.M., Clinton S.K., Durazo-Arvizu R.A., Gallagher J.C., Gallo R.L., Jones G., Kovacs C.S. (2012). IOM committee members respond to Endocrine Society vitamin D guideline. J. Clin. Endocrinol. Metab..

[44] Souberbielle J.C., Body J.J., Lappe J.M., Plebani M., Shoenfeld Y., Wang T.J., Bischoff-Ferrari H.A., Cavalier E., Ebeling P.R., Fardellone P. (2010). Vitamin D and musculoskeletal health, cardiovascular disease, autoimmunity and cancer: Recommendations for clinical practice. Autoimmun. Rev..

[45] Theodoratou E., Tzoulaki I., Zgaga L., Ioannidis J.P. (2014). Vitamin D and multiple health outcomes: Umbrella review of systematic reviews and meta-analyses of observational studies and randomised trials. BMJ.

[46] Pilz S., Tomaschitz A., Drechsler C., Zittermann A., Dekker J.M., Marz W. (2011). Vitamin D supplementation: A promising approach for the prevention and treatment of strokes. Curr. Drug. Targets.

[47] Pilz S., Gaksch M., O’Hartaigh B., Tomaschitz A., März W. (2013). The role of vitamin D deficiency in cardiovascular disease: Where do we stand in 2013?. Arch. Toxicol..

[48] Muscogiuri G., Sorice G.P., Ajjan R., Mezza T., Pilz S., Prioletta A., Scragg R., Volpe S.L., Witham M.D., Giaccari A. (2012). Can vitamin D deficiency cause diabetes and cardiovascular diseases? Present evidence and future perspectives. Nutr. Metab. Cardiovasc. Dis..

[49] Ford J.A., MacLennan G.S., Avenell A., Bolland M., Grey A., Witham M. (2014). Cardiovascular disease and vitamin D supplementation: Trial analysis, systematic review, and meta-analysis. Am. J. Clin. Nutr..

[50] Lim S.S., Vos T., Flaxman A.D., Danaei G., Shibuya K., Adair-Rohani H., Amann M., Anderson H.R., Andrews K.G., Aryee M. (2012). A comparative risk assessment of burden of disease and injury attributable to 67 risk factors and risk factor clusters in 21 regions, 1990–2010: A systematic analysis for the Global Burden of Disease Study 2010. Lancet.

[51] Vimaleswaran K.S., Cavadino A., Berry D.J., Jorde R., Dieffenbach A.K., Lu C., Alves A.C., Heerspink H.J., Tikkanen E., Eriksson J. (2014). Association of vitamin D status with arterial blood pressure and hypertension risk: A mendelian randomisation study. Lancet Diabetes Endocrinol.

[52] Moslehi N., Shab-Bidar S., Mirmiran P., Hosseinpanah F., Azizi F. (2015). Determinants of parathyroid hormone response to vitamin D supplementation: A systematic review and meta-analysis of randomised controlled trials. Br. J. Nutr..

[53] Pilz S., Tomaschitz A., Ritz E., Pieber T.R. (2009). Vitamin D status and arterial hypertension: A systematic review. Nat. Rev. Cardiol..

[54] Kunutsor S.K., Burgess S., Munroe P.B., Khan H. (2014). Vitamin D and high blood pressure: Causal association or epiphenomenon?. Eur. J. Epidemiol..

[55] Beveridge L.A., Struthers A.D., Khan F., Jorde R., Scragg R., Macdonald H.M., Alvarez J.A., Boxer R.S., Dalbeni A., Gepner A.D. (2015). Effect of vitamin D supplementation on blood pressure: A systematic review and meta-analysis incorporating individual patient data. JAMA Intern. Med..

[56] Pilz S., Gaksch M., Kienreich K., Grubler M., Verheyen N., Fahrleitner-Pammer A., Treiber G., Drechsler C., Hartaigh B.O., Obermayer-Pietsch B. (2015). Effects of vitamin D on blood pressure and cardiovascular risk factors: A randomized controlled trial. Hypertension.

[57] Lerchbaum E., Obermayer-Pietsch B. (2012). Vitamin D and fertility: A systematic review. Eur. J. Endocrinol..

[58] Wehr E., Pilz S., Boehm B.O., März W., Obermayer-Pietsch B. (2010). Association of vitamin D status with serum androgen levels in men. Clin. Endocrinol..

[59] Hofer D., Münzker J., Schwetz V., Ulbing M., Hutz K., Stiegler P., Zigeuner R., Pieber T.R., Müller H., Obermayer-Pietsch B. (2014). Testicular synthesis and vitamin D action. J. Clin. Endocrinol. Metab..

[60] Melamed M.L., Michos E.D., Post W., Astor B. (2008). 25-hydroxyvitamin D levels and the risk of mortality in the general population. Arch. Intern. Med..

[61] Schottker B., Haug U., Schomburg L., Kohrle J., Perna L., Muller H., Holleczek B., Brenner H. (2013). Strong associations of 25-hydroxyvitamin D concentrations with all-cause, cardiovascular, cancer, and respiratory disease mortality in a large cohort study. Am. J. Clin. Nutr..

[62] Schottker B., Saum K.U., Perna L., Ordonez-Mena J.M., Holleczek B., Brenner H. (2014). Is vitamin D deficiency a cause of increased morbidity and mortality at older age or simply an indicator of poor health?. Eur. J. Epidemiol..

[63] Thomas G.N., ó Hartaigh B., Bosch J.A., Pilz S., Loerbroks A., Kleber M.E., Fischer J.E., Grammer T.B., Bohm B.O., Marz W. (2012). Vitamin D levels predict all-cause and cardiovascular disease mortality in subjects with the metabolic syndrome: The Ludwigshafen Risk and Cardiovascular Health (LURIC) Study. Diabetes Care.

[64] Pilz S., Marz W., Wellnitz B., Seelhorst U., Fahrleitner-Pammer A., Dimai H.P., Boehm B.O., Dobnig H. (2008). Association of vitamin D deficiency with heart failure and sudden cardiac death in a large cross-sectional study of patients referred for coronary angiography. J. Clin. Endocrinol. Metab..

[65] Zittermann A., Kuhn J., Dreier J., Knabbe C., Gummert J.F., Borgermann J. (2013). Vitamin D status and the risk of major adverse cardiac and cerebrovascular events in cardiac surgery. Eur. Heart J..

[66] Putz-Bankuti C., Pilz S., Stojakovic T., Scharnagl H., Pieber T.R., Trauner M., Obermayer-Pietsch B., Stauber R.E. (2012). Association of 25-hydroxyvitamin D levels with liver dysfunction and mortality in chronic liver disease. Liver Int..

[67] Pilz S., Tomaschitz A., Friedl C., Amrein K., Drechsler C., Ritz E., Boehm B.O., Grammer T.B., Marz W. (2011). Vitamin D status and mortality in chronic kidney disease. Nephrol. Dial. Transpl..

[68] Pilz S., Dobnig H., Tomaschitz A., Kienreich K., Meinitzer A., Friedl C., Wagner D., Piswanger-Solkner C., Marz W., Fahrleitner-Pammer A. (2012). Low 25-hydroxyvitamin D is associated with increased mortality in female nursing home residents. J. Clin. Endocrinol. Metab..

[69] Zittermann A., Iodice S., Pilz S., Grant W.B., Bagnardi V., Gandini S. (2012). Vitamin D deficiency and mortality risk in the general population: A meta-analysis of prospective cohort studies. Am. J. Clin. Nutr..

[70] Pilz S., Iodice S., Zittermann A., Grant W.B., Gandini S. (2011). Vitamin D status and mortality risk in CKD: A meta-analysis of prospective studies. Am. J. Kidney Dis..

[71] Michaelsson K., Baron J.A., Snellman G., Gedeborg R., Byberg L., Sundstrom J., Berglund L., Arnlov J., Hellman P., Blomhoff R. (2010). Plasma vitamin D and mortality in older men: A community-based prospective cohort study. Am. J. Clin. Nutr..

[72] Sempos C.T., Durazo-Arvizu R.A., Dawson-Hughes B., Yetley E.A., Looker A.C., Schleicher R.L., Cao G., Burt V., Kramer H., Bailey R.L. (2013). Is there a reverse J-shaped association between 25-hydroxyvitamin D and all-cause mortality? Results from the U.S. nationally representative NHANES. J. Clin. Endocrinol. Metab..

[73] Chowdhury R., Kunutsor S., Vitezova A., Oliver-Williams C., Chowdhury S., Kiefte-de-Jong J.C., Khan H., Baena C.P., Prabhakaran D., Hoshen M.B. (2014). Vitamin D and risk of cause specific death: Systematic review and meta-analysis of observational cohort and randomised intervention studies. BMJ.

[74] Rejnmark L., Avenell A., Masud T., Anderson F., Meyer H.E., Sanders K.M., Salovaara K., Cooper C., Smith H.E., Jacobs E.T. (2012). Vitamin D with calcium reduces mortality: Patient level pooled analysis of 70,528 patients from eight major vitamin D trials. J. Clin. Endocrinol. Metab..

[75] Wise J. (2014). Nice advises certain groups to take daily vitamin D supplement. BMJ.

[76] Cashman K.D., Kiely M. (2013). EURRECA–Estimating vitamin D requirements for deriving dietary reference values. Crit. Rev. Food Sci. Nutr..

[77] Cashman K.D., Kiely M. (2014). Recommended dietary intakes for vitamin D: Where do they come from, what do they achieve and how can we meet them?. J. Hum. Nutr. Diet.

[78] Ross A.C., Manson J.E., Abrams S.A., Aloia J.F., Brannon P.M., Clinton S.K., Durazo-arvizu R.A., Gallagher J.C., Gallo R.L., Jones G. (2011). The 2011 report on dietary reference intakes for calcium and vitamin D from the Institute of Medicine: What clinicians need to know. J. Clin. Endocrinol. Metab..

[79] Holick M.F., Binkley N.C., Bischoff-Ferrari H.A., Gordon C.M., Hanley D.A., Heaney R.P., Murad M.H., Weaver C.M. (2011). Evaluation, treatment, and prevention of vitamin D deficiency: An Endocrine Society clinical practice guideline. J. Clin. Endocrinol. Metab..

[80] Dawson-Hughes B., Mithal A., Bonjour J.P., Boonen S., Burckhardt P., Fuleihan G.E., Josse R.G., Lips P., Morales-Torres J., Yoshimura N. (2010). IOF position statement: Vitamin D recommendations for older adults. Osteoporos. Int..

[81] Schmid J., Kienreich K., Gaksch M., Grübler M., Raggam R., Meinitzer A., Rutters F., Dekker J.M., März W., Verheyen N. (2013). The importance of assays in vitamin D status classification: A comparison of four automated 25-hydroxyvitamin D immunoassays. LaboratoriumsMedizin.

[82] Binkley N., Krueger D., Cowgill C.S., Plum L., Lake E., Hansen K.E., DeLuca H.F., Drezner M.K. (2004). Assay variation confounds the diagnosis of hypovitaminosis D: A call for standardization. J. Clin. Endocrinol. Metab..

[83] Lips P., Chapuy M.C., Dawson-Hughes B., Pols H.A., Holick M.F. (1999). An international comparison of serum 25-hydroxyvitamin D measurements. Osteoporos. Int..

[84] Carter G.D. (2012). 25-hydroxyvitamin D: A difficult analyte. Clin. Chem..

[85] Sempos C.T., Vesper H.W., Phinney K.W., Thienpont L.M., Coates P.M. (2012). Vitamin D status as an international issue: National surveys and the problem of standardization. Scand. J. Clin. Lab. Investih. Suppl..

[86] Cashman K.D., Kiely M., Kinsella M., Durazo-Arvizu R.A., Tian L., Zhang Y., Lucey A., Flynn A., Gibney M.J., Vesper H.W. (2013). Evaluation of Vitamin D Standardization Program protocols for standardizing serum 25-hydroxyvitamin D data: A case study of the program’s potential for national nutrition and health surveys. Am. J. Clin. Nutr..

[87] Powe C.E., Evans M.K., Wenger J., Zonderman A.B., Berg A.H., Nalls M., Tamez H., Zhang D., Bhan I., Karumanchi S.A. (2013). Vitamin D-binding protein and vitamin D status of black Americans and white Americans. N. Engl. J. Med..

[88] Wahl D.A., Cooper C., Ebeling P.R., Eggersdorfer M., Hilger J., Hoffmann K., Josse R., Kanis J.A., Mithal A., Pierroz A.D. (2012). A global representation of vitamin D status in healthy populations. Arch. Osteoporos..

[89] Hilger J., Friedel A., Herr R., Rausch T., Roos F., Wahl D.A., Pierroz D.D., Weber P., Hoffmann K. (2014). A systematic review of vitamin D status in populations worldwide. Br. J. Nutr..

[90] Calvo M.S., Whiting S.J., Barton C.N. (2005). Vitamin D intake: A global perspective of current status. J. Nutr..

[91] Hintzpeter B., Mensink G.B., Thierfelder W., Müller M.J., Scheidt-Nave C. (2008). Vitamin D status and health correlates among German adults. Eur. J. Clin. Nutr..

[92] Kiely M., Black L.J. (2012). Dietary strategies to maintain adequacy of circulating 25-hydroxyvitamin D concentrations. Scand. J. Clin. Lab. Investig. Suppl..

[93] Romagnoli E., Pepe J., Piemonte S., Cipriani C., Minisola S. (2013). Management of endocrine disease: Value and limitations of assessing vitamin D nutritional status and advised levels of vitamin D supplementation. Eur. J. Endocrinol..

[94] Harvey N.C., Cooper C. (2014). Vitamin D status and ill health. Lancet Diabetes Endocrinol..

[95] Murr C., Pilz S., Grammer T.B., Kleber M.E., Meinitzer A., Boehm B.O., März W., Fuchs D. (2012). Vitamin D deficiency parallels inflammation and immune activation, the Ludwigshafen Risk and Cardiovascular Health (LURIC) study. Clin. Chem. Lab. Med..

[96] Kühn T., Kaaks R., Teucher B., Hirche F., Dierkes J., Weikert C., Katzke V., Boeing H., Stangl G.I., Buijsse B. (2014). Dietary, lifestyle, and genetic determinants of vitamin D status: A cross-sectional analysis from the European Prospective Investigation into Cancer and Nutrition (EPIC)-Germany study. Eur. J. Nutr..

[97] Cashman K.D. (2015). Vitamin D: Dietary requirements and food fortification as a means of helping achieve adequate vitamin D status. J. Steroid Biochem. Mol. Biol..

[98] Black L.J., Walton J., Flynn A., Cashman K.D., Kiely M. (2015). Small increments in vitamin D intake by Irish adults over a decade show that strategic initiatives to fortify the food supply are needed. J. Nutr..

[99] Fulgoni V.L., Keast D.R., Bailey R.L., Dwyer J. (2011). Foods, fortificants, and supplements: Where do Americans get their nutrients?. J. Nutr..

[100] Seamans K.M., Cashman K.D. (2009). Existing and potentially novel functional markers of vitamin D status: A systematic review. Am. J. Clin. Nutr..

[101] Zittermann A., Prokop S., Gummert J.F., Börgermann J. (2013). Safety issues of vitamin D supplementation. Anticancer Agents Med. Chem..

[102] Cashman K.D., Fitzgerald A.P., Kiely M., Seamans K.M. (2011). A systematic review and meta-regression analysis of the vitamin D intake-serum 25-hydroxyvitamin D relationship to inform European recommendations. Br. J. Nutr..

[103] EFSA Panel on Dietetic Products, Nutrition and Allergies (NDA) (2012). Scientific opinion on the tolerable upper intake level of vitamin D. EFSA J..

[104] Davidson M.B., Duran P., Lee M.L., Friedman T.C. (2013). High-dose vitamin D supplementation in people with prediabetes and hypovitaminosis D. Diabetes Care.

[105] Jorde R., Strand Hutchinson M., Kjærgaard M., Sneve M., Grimnes G. (2013). Supplementation with high doses of vitamin D to subjects without vitamin D deficiency may have negative effects: Pooled data from four intervention trials in Tromsø. ISRN Endocrinol..

[106] Tripkovic L., Lambert H., Hart K., Smith C.P., Bucca G., Penson S., Chope G., Hyppönen E., Berry J., Vieth R. (2012). Comparison of vitamin D2 and vitamin D3 supplementation in raising serum 25-hydroxyvitamin D status: A systematic review and meta-analysis. Am. J. Clin. Nutr..

[107] Shab-Bidar S., Bours S., Geusens P.P., Kessels A.G., van den Berg J.P. (2014). Serum 25(OH)D response to vitamin D3 supplementation: A meta-regression analysis. Nutrition.

[108] Autier P., Gandini S., Mullie P. (2012). A systematic review: Influence of vitamin D supplementation on serum 25-hydroxyvitamin D concentration. J. Clin. Endocrinol. Metab..

[109] Zittermann A., Ernst J.B., Gummert J.F., Börgermann J. (2014). Vitamin D supplementation, body weight and human serum 25-hydroxyvitamin D response: A systematic review. Eur. J. Nutr..

[110] Earthman C.P., Beckman L.M., Masodkar K., Sibley S.D. (2012). The link between obesity and low circulating 25-hydroxyvitamin D concentrations: Considerations and implications. Int. J. Obes..

[111] Vimaleswaran K.S., Berry D.J., Lu C., Tikkanen E., Pilz S., Hiraki L.T., Cooper J.D., Dastani Z., Li R., Houston D.K. (2013). Causal relationship between obesity and vitamin D status: Bi-directional Mendelian randomization analysis of multiple cohorts. PLoS Med..

[112] Cianferotti L., Cricelli C., Kanis J.A., Nuti R., Reginster J.Y., Ringe J.D., Rizzoli R., Brandi M.L. (2015). The clinical use of vitamin D metabolites and their potential developments: A position statement from the European Society for Clinical and Economic Aspects of Osteoporosis and Osteoarthritis (ESCEO) and the International Osteoporosis Foundation (IOF). Endocrine.

[113] Sosa M., Láinez P., Arbelo A., Navarro M.C. (2000). The effect of 25-dihydroxyvitamin D on the bone mineral metabolism of elderly women with hip fracture. Rheumatol.

[114] Hoofnagle A.N., Eckfeldt J.H., Lutsey P.L. (2015). Vitamin D-binding protein concentrations quantified by mass spectrometry. N. Engl. J. Med..

[115] Sollid S.T., Hutchinson M.Y., Berg V., Fuskevåg O.M., Figenschau Y., Thorsby P.M., Jorde R. (2016). Effects of vitamin D binding protein phenotypes and vitamin D supplementation on serum total 25(OH)D and directly measured free 25(OH)D. Eur. J. Endocrinol..

[116] Chun R.F., Peercy B.E., Orwoll E.S., Nielson C.M., Adams J.S., Hewison M. (2014). Vitamin D and DBP: The free hormone hypothesis revisited. J. Steroid Biochem. Mol. Biol..

[117] Kupferschmidt K. (2012). Uncertain verdict as vitamin D goes on trial. Science.

[118] Pilz S., Rutters F., Dekker J.M. (2012). Disease prevention: Vitamin D trials. Science.

[119] Kiely M., Cashman K.D. (2015). The ODIN project: Development of food-based approaches for prevention of vitamin D deficiency throughout life. Nutr. Bull..

[120] Halliday G.M., Byrne S.N. (2014). An unexpected role: UVA-induced release of nitric oxide from skin may have unexpected health benefits. J. Investig. Dermatol..

[121] Opländer C., Volkmar C., Paunel-Görgülü A., van Faassen E.E., Heiss C., Kelm M., Halmer D., Mürtz M., Pallua N., Suschek C.V. (2009). Whole body UVA irradiation lowers systemic blood pressure by release of nitric oxide from intracutaneous photolabile nitric oxide derivates. Circ. Res..

[122] Weber K.T., Rosenberg E.W., Sayre R.M. (2004). Suberythemal ultraviolet exposure and reduction in blood pressure. Am. J. Med..

[123] Borradale D.C., Kimlin M.G. (2012). Folate degradation due to ultraviolet radiation: Possible implications for human health and nutrition. Nutr. Rev..

[124] Hassan F.H., Sameer A.S., Ganaie B.A. (2014). Folate: Metabolism, genes, polymorphisms and the associated diseases. Gene.

[125] Slominski A., Wortsman J., Tobin D.J. (2015). The cutaneous serotoninergic/melatoninergic system: Securing a place under the sun. FASEB J..

[126] Watts S.W., Morrison S.F., Davis R.P., Barman S.M. (2012). Serotonin and blood pressure regulation. Pharmacol. Rev..

[127] Lam D.D., Heisler L.K. (2007). Serotonin and energy balance: Molecular mechanisms and implications for type 2 diabetes. Expert. Rev. Mol. Med..

[128] Fonken L.K., Nelson R.J. (2014). The effects of light at night on circadian clocks and metabolism. Endocr. Rev..

[129] Gandini S., Sera F., Cattaruzza M.S., Pasquini P., Picconi O., Boyle P., Melchi C.F. (2005). Meta-analysis of risk factors for cutaneous melanoma: II. Sun exposure. Eur. J. Cancer.

[130] Khalesi M., Whiteman D.C., Rosendahl C., Johns R., Hackett T., Cameron A., Waterhouse M., Lucas R.M., Kimlin M.G., Neale R.E. (2015). Basal cell carcinomas on sun-protected vs. sun-exposed body sites: A comparison of phenotypic and environmental risk factors. Photodermatol. Photoimmunol. Photomed..

[131] Schmitt J., Seidler A., Diepgen T.L., Bauer A. (2011). Occupational ultraviolet light exposure increases the risk for the development of cutaneous squamous cell carcinoma: A systematic review and meta-analysis. Br. J. Dermatol..

